# Sulfate-dependant microbially induced corrosion of mild steel in the deep sea: a 10-year microbiome study

**DOI:** 10.1186/s40168-021-01196-6

**Published:** 2022-01-13

**Authors:** Pauliina Rajala, Dong-Qiang Cheng, Scott A. Rice, Federico M. Lauro

**Affiliations:** 1 Singapore Centre for Environmental Life Sciences Engineering/Nanyang Technological University, 60 Nanyang Dr, Singapore, 637551 Singapore; 2 VTT Technical Research Centre of Finland Ltd., Materials in Extreme Environments, Kemistintie 3, 02044-VTT Espoo, Finland; 3 The School of Biological Sciences, Nanyang Technological University, 50 Nanyang Avenue, Singapore, 639798 Singapore; 4 ithree Institute, The University of Technology Sydney, Ultimo, Australia; 5 Asian School of the Environment, Nanyang Technological University, 50 Nanyang Avenue, Singapore, 639798 Singapore

**Keywords:** MIC, Deep sea, Mild steel, Corrosion, SRB

## Abstract

**Background:**

Metal corrosion in seawater has been extensively studied in surface and shallow waters. However, infrastructure is increasingly being installed in deep-sea environments, where extremes of temperature, salinity, and high hydrostatic pressure increase the costs and logistical challenges associated with monitoring corrosion. Moreover, there is currently only a rudimentary understanding of the role of microbially induced corrosion, which has rarely been studied in the deep-sea. We report here an integrative study of the biofilms growing on the surface of corroding mooring chain links that had been deployed for 10 years at ~2 km depth and developed a model of microbially induced corrosion based on flux-balance analysis.

**Methods:**

We used optical emission spectrometry to analyze the chemical composition of the mooring chain and energy-dispersive X-ray spectrometry coupled with scanning electron microscopy to identify corrosion products and ultrastructural features. The taxonomic structure of the microbiome was determined using shotgun metagenomics and was confirmed by 16S amplicon analysis and quantitative PCR of the *dsrB* gene. The functional capacity was further analyzed by generating binned, genomic assemblies and performing flux-balance analysis on the metabolism of the dominant taxa.

**Results:**

The surface of the chain links showed intensive and localized corrosion with structural features typical of microbially induced corrosion. The microbiome on the links differed considerably from that of the surrounding sediment, suggesting selection for specific metal-corroding biofilms dominated by sulfur-cycling bacteria. The core metabolism of the microbiome was reconstructed to generate a mechanistic model that combines biotic and abiotic corrosion. Based on this metabolic model, we propose that sulfate reduction and sulfur disproportionation might play key roles in deep-sea corrosion.

**Conclusions:**

The corrosion rate observed was higher than what could be expected from abiotic corrosion mechanisms under these environmental conditions. High corrosion rate and the form of corrosion (deep pitting) suggest that the corrosion of the chain links was driven by both abiotic and biotic processes. We posit that the corrosion is driven by deep-sea sulfur-cycling microorganisms which may gain energy by accelerating the reaction between metallic iron and elemental sulfur. The results of this field study provide important new insights on the ecophysiology of the corrosion process in the deep sea.

**Supplementary Information:**

The online version contains supplementary material available at 10.1186/s40168-021-01196-6.

## Introduction

Deep-sea exploration is receiving increasing industrial interest as many resources in easily accessible locations, including minerals, oil, and gas, have been consumed. The advancement of abyssal underwater technologies has opened new possibilities to recover natural resources from deep-sea environments. Even renewable energy applications, e.g., solar, wind, and tidal energy, now look to expand to deep marine areas creating an increasing need for deep water moorings and other infrastructure. The expansion in abyssal exploration requires the use of metallic structures, most commonly steel materials, in the deep sea. However, understanding the behavior of materials under these conditions, an essential component for environmentally safe and economically feasible operations, remains limited.

The corrosion process, which plays a detrimental role in the overall longevity of subsea structures, has been well studied under low hydrostatic pressure in surface and shallow marine waters [1–3]. However, the deep-sea environment is very different from the shallow marine areas and is characterized by low temperature, limited and sporadic inputs of organic nutrients, and high hydrostatic pressure. There is an increasing body of evidence that corrosion mechanisms of steel under high hydrostatic pressure differs from those in shallow waters [4–8]. The increasing hydrostatic pressure appears to accelerate the initiation of metastable pitting and decreases the probability of pit growth, which increases the susceptibility of steel materials to uniform corrosion [4]. The metallic structures in deep-sea applications are in contact with the natural environment and thus, they are susceptible to microbially induced corrosion (MIC) and biofouling. Deep-sea sediment contains microorganisms (bacteria, archaea, fungi, and protists), which may accelerate corrosion in environments where corrosion would not otherwise be favorable. In particular, microorganisms may greatly contribute to the corrosion of steel because they are able to accelerate several types of corrosion, including both generalized and localized corrosion (e.g., pitting, crevice corrosion, and stress corrosion cracking) [9–11]. Furthermore, it is well-known that microbial metabolic activity, diversity, physiology, and ecology are strongly impacted by hydrostatic pressure [12, 13]. Thus, it is essential to investigate pressure-adapted microorganisms in the context of MIC.

Typically, uncoated mild steel corrodes rapidly in seawater, developing a corrosion product layer that acts as a diffusion barrier, protecting the steel from further corrosion. However, microbial communities formed on steel surfaces are known to destabilize this corrosion product layer, thus, allowing the corrosion to continue [14]. Abiotically produced hydrogen in cathodic corrosion reactions may act as a chemoattractant for microorganisms natively present in deep-sea environment [15]. One such group of microorganisms is the sulfate reducing bacteria (SRB). In seawater environments, SRB are known to accelerate the overall corrosion process of mild steel [16–19] and to contribute to the localized corrosion damage [20] and stress corrosion cracking [10, 21, 22] of the steel.

Few studies have investigated the mechanisms of MIC under deep-sea conditions. However, understanding the role of microorganisms and the mechanisms by which they induce corrosion is important to accurately determine the operational life-time of infrastructure in deep-sea settings and to mitigate risk to deep water installations. In this study, the role of microbial communities on the corrosion of steel mooring chain links that were exposed to deep-sea conditions for ten years is presented.

## Materials and methods

### Sampling

**Fig. 1:**
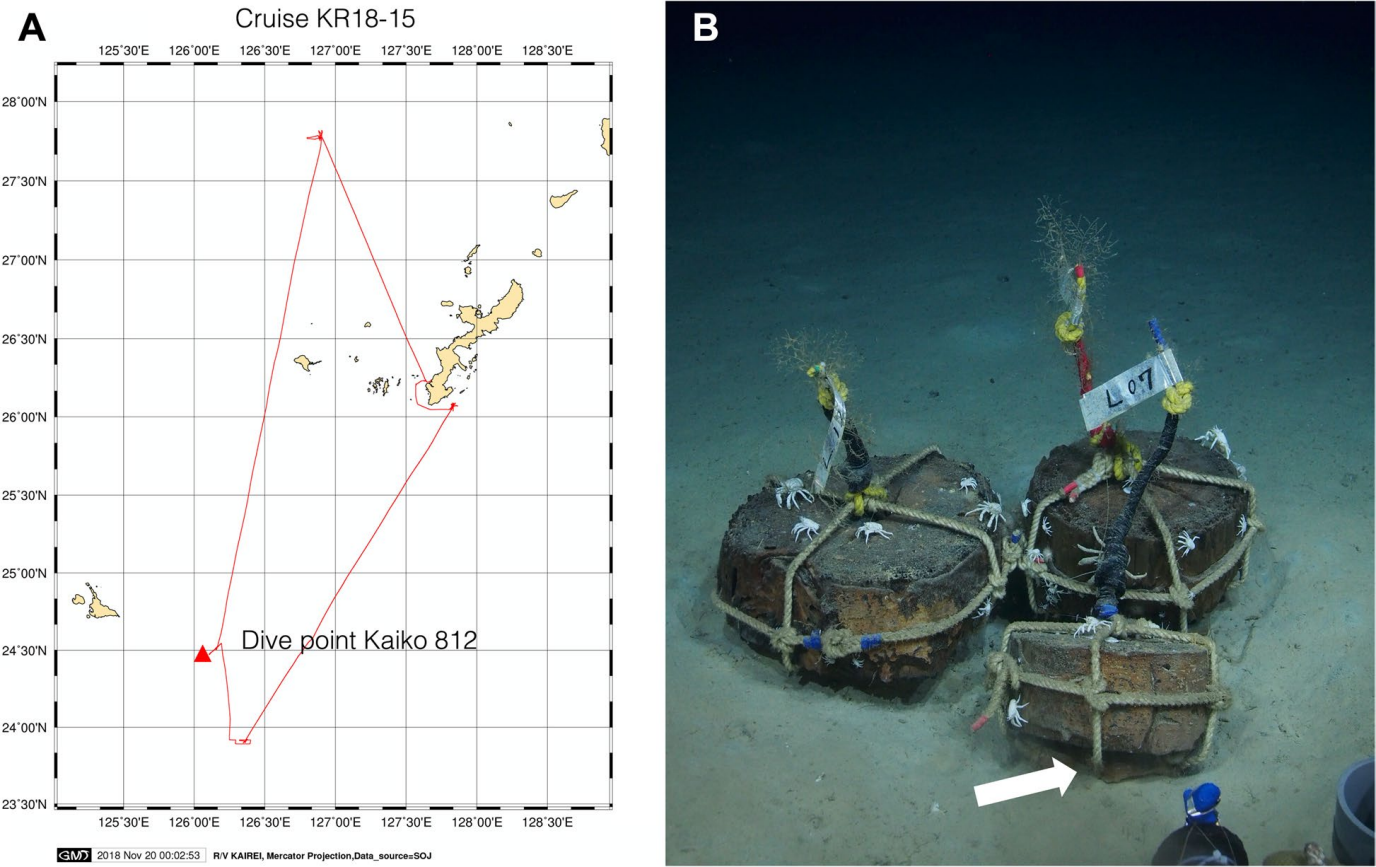
**A** Map of the study site, indicating the KR18-15 cruise route and dive point of the KAIKO 812 dive to collect the mooring chain links after 10 years of deployment. **B** Image of the mooring chain location immediately before retrieval (white arrow). JAMSTEC (2019) KAIREI KR18-15 Cruise Data. JAMSTEC. doi:10.17596/0001281 (accessed 2020-12-20)

Mooring chain links, one full and three half-links, were first deployed in June 2008 as part of expedition NT08-12, R/V Natsushima using ROV Hyperdolphin (dive HDP857) at coordinates 24° 31.3′ N, 126° 09.9′ E, and at a depth of 1988 m. Mooring chain links were retrieved 10 years later, in November 2018, during R/V Kairei expedition KR 18-15 using ROV Kaiko (dive 812) (Fig. 1A and B). Mooring chain links were retrieved to R/V Kairei in sealed boxes to protect them from surface water contamination. Three half-links were reserved for molecular biological studies and subsequent metallurgical studies and one full link was reserved for surface characterization. The dimensions of the full link were 10 cm (w) and 17 cm (h) and the half links were cut to 6.5 cm (h). After retrieval, the mooring chain links were immediately photographed and stored in sterile zip lock bags and placed in a −80 °C freezer until transported to an on-shore laboratory (the links reserved for molecular biological studies) or dried under N_2_ flow and stored in zip lock bags and in a −80 °C freezer until the corrosion products were collected for more detailed studies.

A push core sediment sample was also collected from a location immediately beneath the mooring chain links (Fig. 1B). The sediment core was dissected into subsamples representing the following depths 2‑4 cm, 4‑7 cm, and 7‑9 cm below the sediment surface. The surface layer, 0‑2 cm, was discarded due to possible contamination during sampling. The sediment subsamples were stored at −80 °C until DNA extraction. The environment was characterized by cold seawater (2 °C), with low dissolved oxygen (2 mg/L) and a salinity of 3.5% and high hydrostatic pressure (~20 MPa, 200 bar).

### Surface characterization

The elemental composition of the mooring chain steel was determined using optical emission spectrometer ARL iSpark 8860 (Thermo Fisher Scientific, MA, USA). The corrosion product formed on mooring chain surfaces was analyzed with an energy-dispersive X-ray spectrometer (EDS) coupled with a scanning electron microscope (SEM) (SEM ULTRAplus, Carl Zeiss, Oberkochen, Germany). Crystalline corrosion products were further identified with an X-ray diffraction spectrometer (XRD) (Empyrean, PANalytical B.V., Almelo, Netherlands).

The form of corrosion on surfaces and in cross-section specimens was studied from the same three links that were also subjected to microbial analyses. After removal of biomass (the “DNA extraction” section), the remaining residuals of corrosion product were removed according to ASTM standard G 1–90, method C 3.1 [23]. The cross-section specimens were imaged using optical microscopy after polishing and subsequent etching with Nital. To visualize the morphology of surface deposits and possible biofilm formed on the surface, scrapings of surface deposits of individual mooring chain links were fixed in a phosphate solution (0.1 M, pH 7.2) buffered with 2.5% glutaraldehyde for 12 h and then were dehydrated with an ethanol series from 30%, 50%, 75%, 80%, 96% to absolute, followed by a final drying in hexamethyldisilazane (Merck, Germany). The samples were coated with Pd (10–15 nm) prior to FE-SEM examination (SEM ULTRAplus, Carl Zeiss, Oberkochen, Germany) operated at 1 to 5 kV.

### DNA extraction

The biomass for subsequent molecular biological analyses was extracted from the mooring chain surfaces by first scraping the outer corrosion product layer and subsequently bead-beating the mooring chain links in 100 mL sterile phosphate-buffered saline (PBS) solution and Tween20 (Merck, Germany) (1 μL of Tween20 per 1 mL PBS) for 20 min at 150 rpm agitation. The link samples were then ultra-sonicated for 3 × 30 s to remove the inner, tightly attached deposit layer. The extracted biomass was finally collected on a 0.22-μm pore-size polyethersulfone filter membrane (Corning, MA, USA) for DNA extraction. The membranes were cut out of the filtration units with sterile scalpel blades and stored at −80 °C. DNA was extracted from the biomass collected on filters (mooring chain) (0.2 g) or sediment samples (0.5 cm^3^) stored at −80 °C using the DNeasy PowerSoil Isolation kit (Qiagen, Germany) in accordance with the manufacturer’s protocol and eluted into 50 μL of the elution buffer supplied by the manufacturer. No-sample, negative extraction control reactions were prepared alongside the samples. DNA was quantified using a Qubit® 2.0 fluorometer in combination with the dsDNA Broad Range assay kit (Invitrogen, Singapore).

### Quantitative PCR

Quantitative polymerase chain reaction (qPCR) was used to determine the number of genes of bacterial or archaeal 16S rRNA and SRB by the β-subunit of the dissimilatory sulfite reductase (*dsrB*) on mooring chain surfaces to estimate cell numbers. The primers used in the qPCR were UniFR334 and UniFR514 for bacterial 16S rRNA gene [24], ARC344f, and A744R for archaeal 16S rRNA gene [25, 26], and DSRp2060F and DSR4R for the *dsrB* gene [27, 28]. An artificial plasmid containing the corresponding gene ranging from 10^2^ to 10^8^ copies per reaction was used as an external standard.

The qPCR was performed in triplicate 10 μL reactions for each sample and a standard dilution using the KAPA SYBR® FAST qPCR Kit Master Mix (2×) ABI Prism™ (Kapa Biosystems, MA, USA). The reactions contained 1 μL template DNA, standard dilution, or PCR grade water for the negative control reactions and 2.5 pmol of each primer. The qPCR assays were performed on a StepOnePlus™ real-time PCR system (Applied Biosystems, CA, USA). The amplification program consisted of an initial denaturation step of 15 s at 95 °C followed by 45 amplification cycles consisting of a denaturation at 95 °C for 10 s, annealing at 58 °C and an extension step of 30 s at 72 °C, with fluorescence measurement at the end of each extension. The amplification cycles were followed by a final extension for 3 min at 72 °C, and melting curve analysis. For the melting curve analysis, the amplicons were first denatured at 95 °C, followed by an annealing step at 60 °C and a progressive denaturation to 95 °C with a temperature increase of 2.2 °C/s and continuous fluorescence measurement.

### Amplicon library preparation and sequencing

Amplicon libraries for sequencing were generated using a modified version of the Illumina 16S Metagenomic Sequencing Library Preparation Protocol from the mooring chain surface samples and the sediment samples. The universal primer pairs (926wF: 5′-AAACTYAAAKGAATTGRCGG-3′ and 1392R: 5′-ACGGGCGGTGTGTRC-3′) targeting the V6-V8 hyper-variable region of the 16S/18S ribosomal RNA gene with an Illumina-specific overhang were used [29]. For each sample, triplicate PCR reactions obtained with 22 cycles were pooled and purified using Agencourt AMpure XP beads (Beckman Coulter, Singapore). Products from this first step were then sequenced by the Singapore Centre for Environmental Life Sciences Engineering (SCELSE) where a second round of PCR was performed to add dual barcodes to each amplicon library. The raw amplicon sequences were deposited in the Sequence Read Archive (SRA) of the National Center for Biotechnology Information under BioProject number PRJNA693108.

### Metagenomic sequencing

Metagenomic sequencing of the microbial communities on the mooring chain surfaces was conducted at the SCELSE sequencing facility on a MiSeq platform (Illumina) using the low input protocol previously described [30]. Prior to library preparation, the quality of the DNA samples was determined using Agilent 2200 TapeStation System for genomic DNA. The raw metagenomic sequences were deposited in the Sequence Read Archive (SRA) of the National Center for Biotechnology Information under BioProject number PRJNA693108.

### Sequence analysis

#### Amplicon sequence analysis

Microbial amplicon sequences were analyzed using Qiime2-DADA2 v. 2020.8.0 pipeline [31, 32]. The “dada2 denoise-paired” method was used for detecting and correcting Illumina amplicon sequences. In this process, quality filtering was also performed. Parameters “—p-trim-left-f 5” and “—p-trim-left-r 5” were used to trim off the first 5 bases of both forward and reverse reads. Parameter “—p-trunc-len-f 280” was used to truncate the forward sequences at position 280. Parameter “—p-trunc-len-r 240” was used to truncate the reverse sequences at position 240. Sequences were classified using “feature-classifier classify-sklearn” method with parameter “—i-classifier silva-138-99-nb-classifier.qza.” The taxonomic composition of each sample was illustrated using the “taxa barplot” method. The alpha- and beta-diversity metrics were generated using the “core-metrics-phylogenetic” method. The associations between categorical metadata and alpha diversity data (the Shannon’s diversity index, Faith’s Phylogenetic Diversity and Evenness) were tested using “diversity alpha-group-significance” method. A principal coordinate analysis (PCoA) plot was generated using “diversity beta-group-significance” and “emperor plot” methods based on the unweighted UniFrac distance metrics [33].

#### Metagenomic sequence analysis

Metagenomic sequences (6 samples for a total of 1,5320,938 paired-end sequencing reads) were filtered and trimmed using customized Perl scripts. Reads containing a minimum of 80% of the bases with quality score of 20 or higher were retained and the others were discarded. The reads were trimmed from right to left at the bases with a minimum quality score of 25. After trimming, only reads containing minimum length of 30 were kept. Quality passed paired reads were used for subsequent analyses. Metagenomic data was binned using Anvi’o v. 6.2 [34]. All of the quality-filtered reads from the 6 samples were combined, followed by assembly using MEGAHIT v. 1.2.9 [35]. Assembled contigs were checked using “anvi-script-reformat-fasta” with parameter “–min-len 500.” A BAM alignment file for each sample was generated using Bowtie2 [36] and Samtools [37] and “anvi-init-bam.” An anvi’o contig database was generated using “anvi-gen-contigs-database” and open reading frames were identified using Prodigal v. 2.6.3 [38]. The profile of each BAM file was linked to the contigs database using “anvi-profile” with parameter “—min-contig-length 2500.” All profiles were then merged using “anvi-merge.” Taxonomy was assigned to contigs using Kaiju v. 1.7.3 [39]. The kaiju taxonomic profile was imported into the contig database using “anvi-import-taxonomy-for-genes.” The binned metagenome-assembled genomes (MAGs) were then clustered using the anvi’o interactive interface “anvi-interactive” based on hierarchical clustering, taxonomic identity, and GC content. The completeness and contamination of each identified MAG bin were determined using CheckM v. 1.1.2 [40]. Based on CheckM results, bins were manually refined using “anvi-refine.’ The final MAG bins were assessed for completeness and contamination using CheckM again. Bins were defined as high-quality draft (> 90% complete, < 5% contamination), medium-quality draft (> 50% complete, < 10% contamination), or low-quality draft (< 50% complete, < 10% contamination) MAGs. Taxonomy was assigned to MAGs using the GTDB-Tk v. 1.3.0 software [41] based on release 95 data. The phylogenetic trees of MAGs from GTDB-Tk results were displayed using FigTree v. 1.4.4 (http://tree.bio.ed.ac.uk/software/figtree/) (Supplementary Fig. S1).

The percent recruitment of each bin from different samples was obtained from Anvi’o using “anvi-summarize.” These percent recruitment values can be read as “X percent of all mapped reads in Sample Y mapped to splits that were binned into bin Z.” For each bin, a paired sample *t* test was calculated to compare the percent recruitment difference between paired samples from the inner and the outer layer of each mooring chain. A principal coordinate analysis (PCoA) with Bray-Curtis dissimilarity using the percent recruitment matrix was performed using the ape [42] and vegan [43] libraries on R.

#### Recovery of genes involved in thiosulfate metabolism and iron metabolism

The seed sequences from TsdA (ADC61061.1), SoxB (CAA55824.2), and Sdo (WP_009565301.1) were used to perform a Blast [44] search against the NCBI non-redundant protein database. The blast-hits were aligned using MUSCLE [45] and each alignment was used to generate an HMM profile with HMMER v. 3.3.1 (http://hmmer.org/). The profile was searched against each binned MAG. Hits with an *e* value smaller than 0.001 were considered as a recovered gene for *tsdA*, *soxB*, and *sdo*, respectively. Iron metabolism related genes were searched using the FeGenie software [46]. FeGenie created its own database of HMMs based on genes related to iron acquisition, regulation, storage, reduction/oxidation, and magnetosomes in bacteria and archaea.

#### Identifying hallmarks of deep-sea adaptation

Blastp was used to search the reference phrB gene (WP_001142014.1) against the UniProtKB/Swiss-Prot database [47]. Sequences annotated as “Deoxyribodipyrimidine photo-lyase” from blastp hits were selected and aligned. An HMM profile for the alignment was generated and searched against the annotated protein databases using the HMMER software (hmmer.org). The predicted proteome of the metagenomes were generated with two different methods: MetaGeneMark [48] and PROKKA [49]. The MetaGeneMark annotated proteins identified three candidate genes related to *phrB*, although these had very low coverage and did not belong to any binned MAGs. The PROKKA annotated proteins did not recover any putative *phrB* candidates.

Transposable elements (TE) were identified using RepeatModeler package [50]. The dfam database (dfam.org) incorporated into the RepeatMasker (repeatmasker.org) tool was used for identification. The number of TEs for each MAG was normalized the sequence length and compared to the normalized numbers from the closest complete genome from shallow water. The lengths of intergenic regions were compared between the deep-sea isolate *Desulfocapsa* sp. MC-01 and its closest shallow water isolate *Desulfocapsa sulfexigens* DSM 10523. Customized Perl scripts were used to calculate the lengths of intergenic regions based on annotated genomes. Annotated information of *D. sulfexigens* DSM 10523 was from GenBank, while the annotated information of *Desulfocapsa* sp. MC-01 was from the PROKKA method.

#### Metabolic modeling based on MAGs

A microbial metabolic model was constructed using the KBase webserver [51]. The FASTA file for each binned MAG was imported in the pipeline and each MAG was annotated using the RASTtk v. 1.073 toolkit [52]. A draft metabolic model was built using the ModelSEED app [53] with gap-filling and assuming complete media. Flux-balance analysis (FBA) was then performed on the default complete media [54]. All MAGs with completeness > 80% were subjected to the FBA analyses separately and all FBA models were merged into a compartmentalized community model using “construct mixed-bag model.” The community model was analyzed by FBA to predict metabolic fluxes. The final metabolic model was visualized on the KEGG pathway using the iPATH webserver [55].

## Results

### Corrosion

The composition of the mooring chain steel was (by %w/w) C 0.28%, Si 0.24%, Mn 1.47%, S 0.015%, P 0.017%, Cr 0.15%, Ni 0.04%, Mo 0.02%, Cu 0.09%, Al 0.014%, V 0.005%, Ti 0.002%, Co 0.008%, Nb 0.002%, Sn 0.005%, As 0.005%, and Fe to balance, corresponding to the composition of mild steel grade QR3. Upon retrieval, the mooring chain links were completely covered with thick black surface deposit with a distinct smell of hydrogen sulfide. The EDS result (Supplementary Data S1) indicated a high sulfur (5‑25 w%) content in the corrosion product layer. Other major components observed by EDS were iron (Fe 8‑62 w%), oxygen (O 30‑52 w%), and carbon (C 5‑19 w%). In addition to these major components, Si, Na, Mg, Al, Cl, K, Ca, and Mn were also detected. XRD analysis of the crystalline fraction of the corrosion products indicated the presence of magnetite (Fe_3_O_4_), FeCO_3_, and iron(II) sulfide (FeS) and the majority of corrosion products were amorphous.

**Fig. 2:**
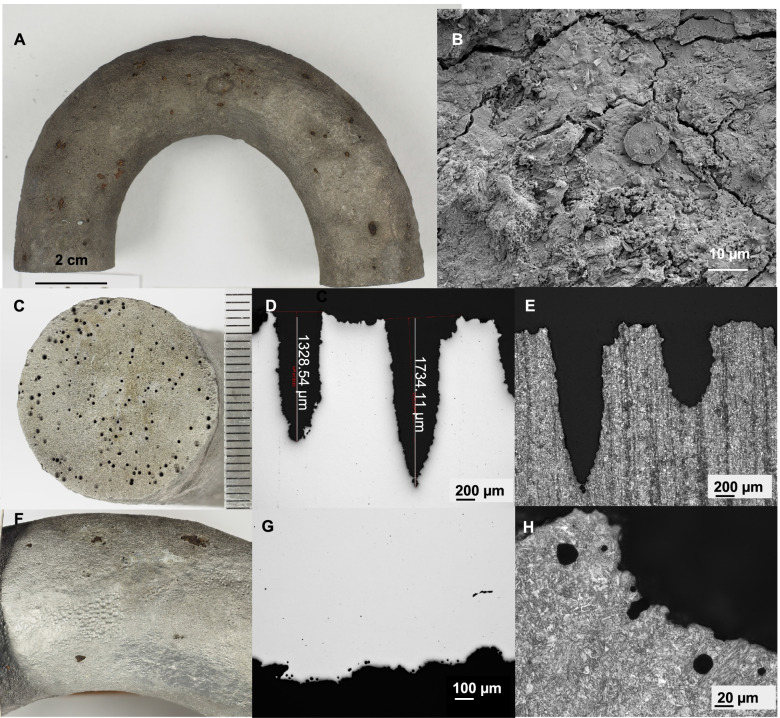
Mooring chain links demonstrating heavy localized corrosion after 10 years of exposure to deep-sea conditions. **A** General overview of a chain link after removal of the corrosion products. **B** SEM image of the corrosion products. **C** Intensive pitting observed at the ends of the chain link. **D**‑**E** Cross-section analyses of pitting penetration to the base material on the ends of mooring chain link, polished and etched. **F** Wide, localized corrosion on the chain link sides. **G**‑**H** Cross-section analyses of pitting penetration to the base material, polished, and etched surface

Removal of the deposit layer revealed intensive, localized corrosion (Fig. 2A, C). The general corrosion on the mooring chain surface, based on cross section measurements, was estimated to be ~100 μm/year. The localized corrosion rate was higher than the general corrosion rate, with some pits being up to 2-mm deep on the ends of mooring chain link, giving an estimated corrosion rate up to 200 μm/year (Fig. 2D and G).

The microstructure of the mooring chain links was typical for alpha ferritic steel (Fig. 2E and H). Banding was visible on the vertical cross sections (Fig. 2E and H). The pitting penetration depth was greater at the end of mooring chain links along the banded microstructure (2 mm) than on the sides of the links (200 μm) (Fig. 2D and G). The pitting on the side of the links was wider (up to 4-mm width) (Fig. 2F, G, H) but occasionally reached the subsurface of steel forming tunnels, whereas the pitting was narrow and deep at the ends of links (Fig. 2C, D, E).

### Microbial community

SEM imaging of random surface sections revealed extensive colonization by microbial cells on the outer surface of the mooring chain as well as coccolithophore plates and fragments (Fig. 2B). Bacterial and archaeal communities from the inner and outer deposit layers of the mooring chain were quantified using qPCR. For all three mooring chains, the bacterial community was more numerous in the inner layer than the outer, 10^9^ and 10^5^‑10^7^ bacterial 16SrRNA gene copies/g, respectively (Supplementary Fig. S2). Sulfate reducing bacteria, according to *dsrB* gene copy number, were up to 3 log more numerous in the inner layer (10^9^ gene copies/g) than in the outer layer (10^5^‑10^6^ gene copies/g) as well (Supplementary Fig. S2).

**Fig. 3:**
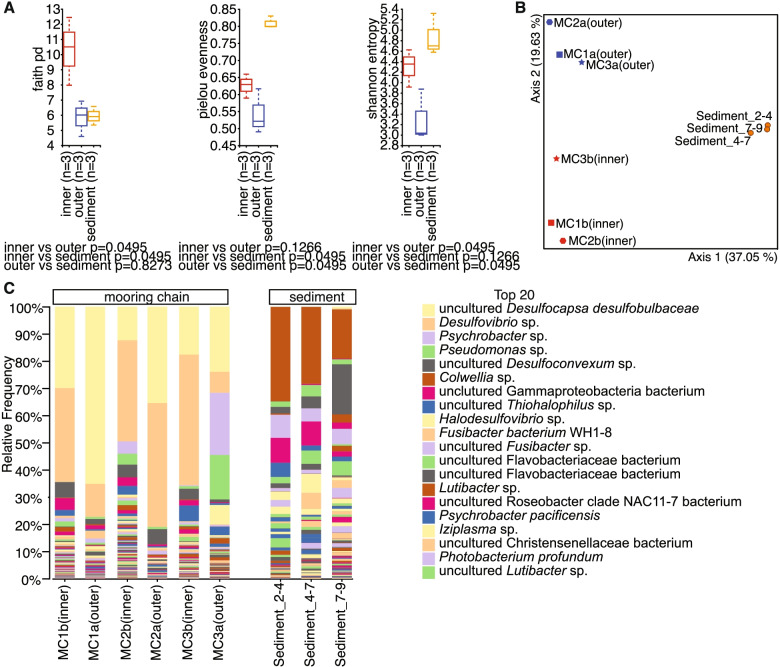
**A** Alpha diversity metrics calculated based on Shannon’s diversity index, Faith’s phylogenetic diversity, and evenness based on 16S rRNA amplicon sequencing. Red refers to the mooring chain inner layer, blue refers to the mooring chain outer layer, and orange refers to the sediment. **B** Microbial community differences as visualized with PCoA using the unweighted UniFrac distance metrics. Axes 1 and 2 explain 37.05% and 19.63% of the variance, respectively. Red refers to the mooring chain inner layer, blue to the mooring chain outer layer and orange to the sediment. **C** The relative abundance of microbial species detected on the mooring chain inner and outer layers or in the sediment (depths 2‑4 cm, 4‑7 cm, and 7‑9 cm)

**Fig. 4:**
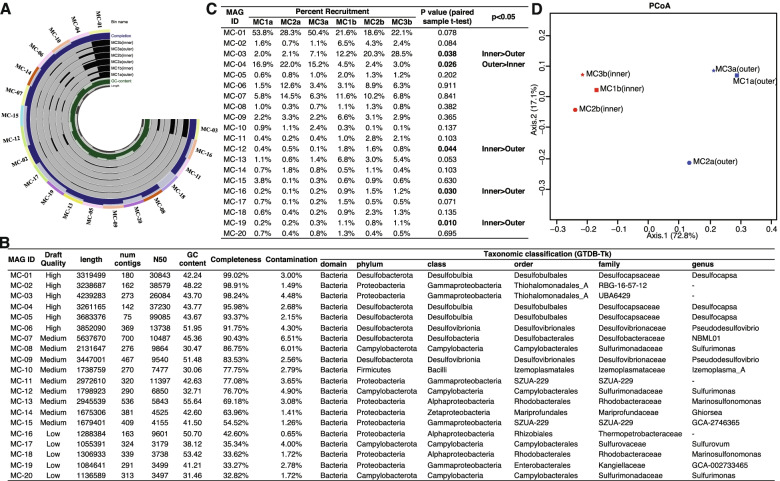
**A** Comparison of binned MAGs across samples comparing mean coverage, genome completion, GC-content, and genome length. The summary was generated and visualized using “anvi-summarize.” **B** Genomic feature summary and taxonomic identification of MAGs in the microbial community from the deep-sea mooring chain link corrosion products. Genomic features are summarized below for each MAG including draft quality, length, number of contigs, N50, percent GC content, completeness, and contamination estimated by CheckM. Putative taxonomies were identified with GTDB-Tk including 7 main ranks (domain, phylum, class, order, family, genus, and species). The species columns were all null and not shown. MAGs are sorted by percent completeness. (**C**) Comparison of the percent recruitment of each MAG between samples. For each bin, a paired sample *t* test was calculated to compare the percent recruitment difference between paired samples from the inner and the outer layer of each mooring chain. Bold text indicates a statistically significant difference with a *p* value less than 0.05. **D** PCoA plot with Bray-Curtis dissimilarity using the percent recruitment matrix. Result revealed that the inner and outer samples clustered separately. Axis 1 explains 72.8% of the variance. Red refers to the mooring chain inner layer and blue refers to the mooring chain outer layer

The microbial community was characterized based on 16S rRNA amplicon sequencing and metagenomic sequencing. The microbial community on the inner layer was more diverse than outer layer (Fig. 3A, B). Both methods used to characterize the composition of the microbial community (i.e., amplicon metabarcoding and metagenomic) depicted a similar taxonomic structure of the microbiome. The biofilm formed on the surface of the mooring chain link was dominated by sulfur cycling bacteria of the genera *Desulfocapsa* (10‑65% of the microbial community) and *Desulfovibrio* (10‑45% of the microbial community) (Figs. 3C and 4). *Desufocapsa* and *Thiohalophilus* were the most abundant genera on the outer layer of the corrosion deposit, and *Desulfovibrio* were more abundant on the inner layer. The microbial community in the sediment layers from 2 to 9 cm differed from that detected on mooring chain surfaces and was dominated by bacteria belonging to genus *Colwellia*, an unclassified Archaeal genus of *Nitrosopumilaceae* and an unclassified genus of *Flavobacteriaceae* (Fig. 3C).

### Flux-based analysis of microbiome metabolisms

The community metabolic model was established by merging 9 individual metabolic models (with MAG completeness > 80%). FBA results showed that the objective value of the merged community model (representing the maximum achievable flux through the biomass reaction of the community metabolic model) was 53.68, which indicated that the microbial community could grow well based on the complete media and gap-fill models. Individually, the objective value of 8 metabolic models were lower than for the merged model and ranged from 6.42 to 45.17 (with the exception MC-09 as 56.46), which indicated that the microbial community as a whole could grow faster than each individual taxon grown separately (Supplementary Table S1).

**Fig. 5:**
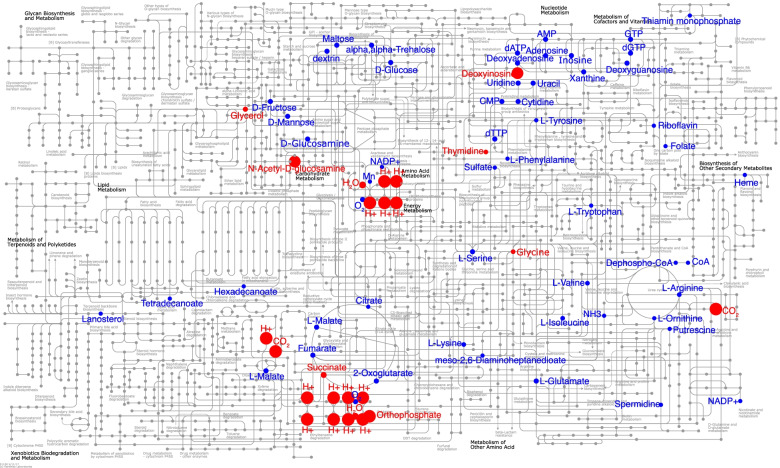
FBA visualization of the uptake/secretion of metabolites using iPATH. Positive uptake from the extracellular compartment is highlighted in blue. Negative uptake from the extracellular compartment is highlighted in red. The dot size is proportional to the absolute value of the rate of uptake/secretion. The community metabolic model was generated by merging 9 separate metabolic models (with MAG completeness > 80%). The highest secretion rates were observed for H^+^ and CO_2_, most likely because of their role in oxidative phosphorylation, methane metabolism, energy metabolism, and urea cycling. Sulfate uptake was observed as part of the sulfur metabolism

Metabolites H^+^ and CO_2_, which had the highest secretion rates, are likely the products of multiple metabolic pathways, including oxidative phosphorylation, methane metabolism, energy metabolism, or the urea cycle. With respect to sulfur metabolism, a significant uptake of sulfate was also predicted by the model. All these metabolites are related with reactions that may potentially cause MIC as further discussed below (Fig. 5).

## Discussion

The corrosion rate of mooring chain steel detected here was higher than could be expected based on chemical conditions alone. It has been estimated that the corrosion of steels and iron in the deep sea is directly proportional to the amount of dissolved oxygen in seawater [56]. The dissolved oxygen in seawater close to the mooring chain deployment location was 2 mg/L, which would result in an average corrosion rate of 75 μm/year according to Reinhart [56], a value much lower than the ~100 μm/year estimated in the present study. It is reasonable to assume that the actual oxygen concentration on the mooring chain surface was even lower as it was partly embedded in the sediment, which would result in a lower corrosion rate based only on abiotic reactions. Moreover, abiotically driven corrosion under deep-sea conditions is expected to be uniform [56], whereas in the present study, the corrosion was highly localized. In some cases, the steel microstructure (e.g., banding) is associated with an increased tendency for localized corrosion, namely, end-grain corrosion, explaining the deeper penetration at the ends of the links compared to the sides of links [57–59].

Typically, large iron oxides/hydroxides dominate structures referred to as rusticles, which are observed for ship wrecks or sunken submarines, are commonly thought to be the product of iron oxidizing bacteria and SRB [60–62]. The deep-sea corrosion related to ship wrecks typically takes place several meters to tens of meters above the sea floor in open water bodies. In contrast, the mooring chain links were embedded in sediment where the environment is likely micro-oxic/anoxic resulting in a very different MIC process. Thus, the corrosion process observed in this study was very different from most reported cases of deep-sea MIC and this may be correlated to the distinct community composition and environment of this study.

The microbiome of the mooring chain biofilm was dominated by SRB, both according to *dsrB* gene abundance as well as amplicon and metagenomic community analyses. The microbial community in the surrounding sediments differed greatly from the community enriched on mooring chain surfaces. This was especially the case for sulfur or thiosulfate disproportionating *Desulfocapsa* and sulfate reducing *Desulfovibrio* or *Pseudodesulfovibrio* species that were enriched on the mooring chain steel surface. They may benefit from the interaction with the abiotic corrosion process where H_2_ and Fe^2+^ are released. *Desulfocapsa* detected within the mooring chain corrosion product resembles species that are disproportionate inorganic sulfur compounds, S^0^ or thiosulfate, and that grows autolithotrophically fixing CO_2_ [63]. Disproportion of elemental sulfur is not energetically efficient if sulfide accumulates unless an external oxidant, in this case Fe^2+^, is present to act as a sulfide scavenger, which makes the process energetically favorable, often resulting in the accumulation of FeS or FeS_2_ [64, 65]. In support of this mechanism, FeS was identified as one of main components forming the surface deposit layer on the mooring chains. The absence of genes characteristic of shallow-water SRBs, such as the deoxyribodipyrimidine photo-lyase gene involved in the repair of UV radiation-induced DNA damage, suggests that the species involved in the corrosion process are indeed true deep-sea species [66]. Interestingly, other genomic features previously associated with microbial life in the deep-sea such as larger intergenic regions (Supplementary Fig. S3) and a higher proportion of transposable elements (Supplementary Table S2) [13] were not observed in the MAGs of this study. Whether this relates to habitat specific differences in gene proliferation, population bottlenecks or the unique ecological interactions occurring within the biofilm of a mooring chain remains to be discovered.

In addition to sulfur cycling microorganism, other cold, deep-sea high hydrostatic pressure adapted microorganisms were detected in lower abundance on the mooring chain surface. These included Fe^2+^ or H_2_ oxidizing microorganisms, such as bacteria belonging to the *Mariprofundus* genus that might also benefit from abiotic corrosion processes [67]. Interestingly, these taxa have often been linked to the production of iron oxyhydroxides that were not detected here. This might suggest that either these microorganisms are not actively oxidizing Fe^2+^ under these conditions or that their metabolic end products are readily, and abiotically, reduced to FeS.

Consistent with the hypothesis that the cycling of iron in this system is entirely abiotic, only iron transporters and siderophores were found in the MAG analysis (Supplementary Table S3). This would suggest that iron was used for assimilation but not for energy-generation. The assimilatory uptake of iron is also supported by the observation of positive uptake of heme in the community metabolic model (Fig. 5).

Sulfate can be reduced by SRB (e.g., *Desulfovibrio* and *Pseudodesulfovibrio* detected here) utilizing cathodic or biologically produced H_2_ (Fig. 5) as the electron donor by the following series of reactions:

1 $${\mathrm{Fe}}^0+{2\mathrm{H}}^{+}\to {\mathrm{Fe}}^{2+}+{\mathrm{H}}_2$$

2 $${{\mathrm{SO}}^{2-}}_4+{\mathrm{H}}^{+}+{4\mathrm{H}}_2\to {\mathrm{H}\mathrm{S}}^{-}+{4\mathrm{H}}_2\mathrm{O}$$

**Fig. 6:**
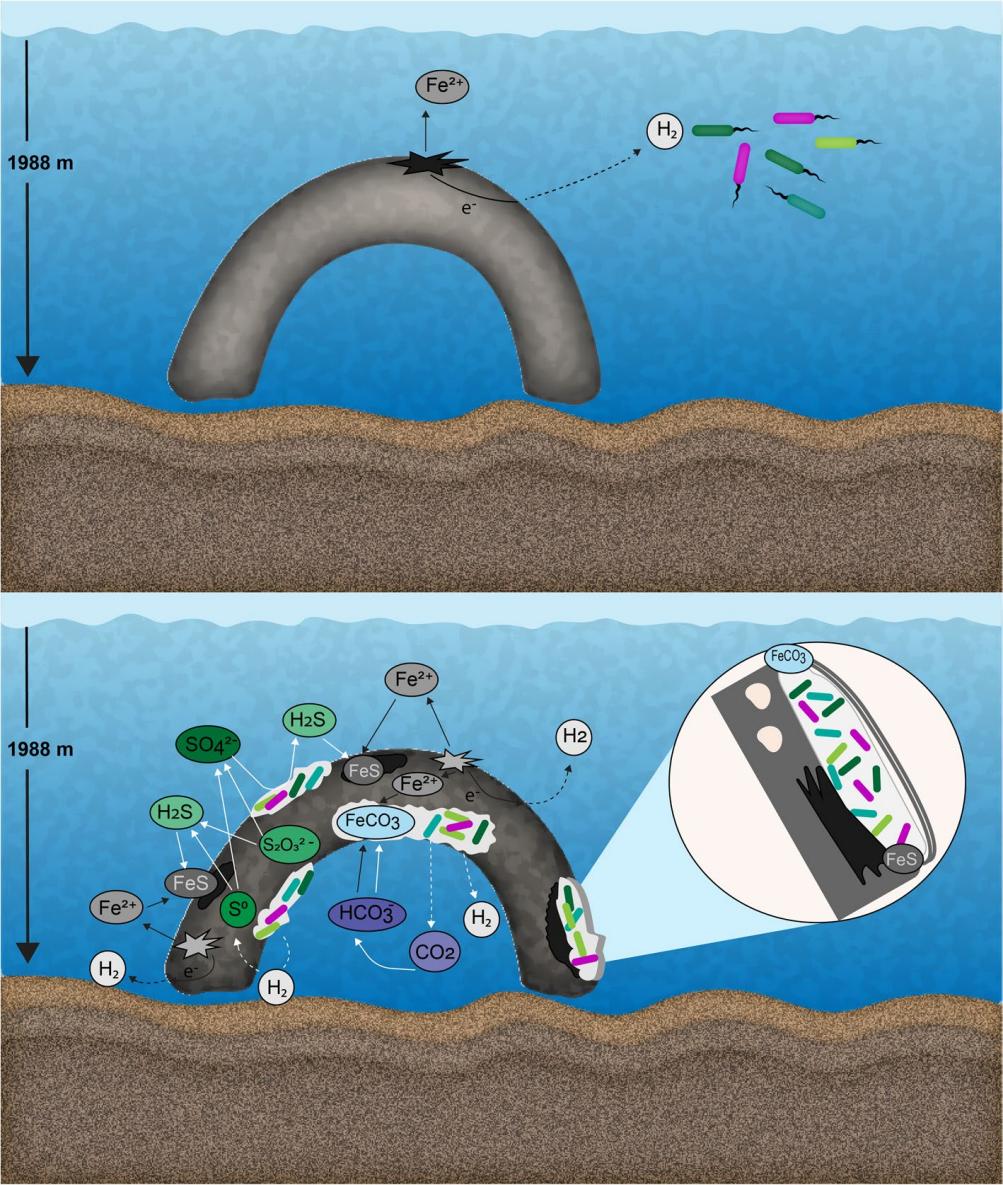
Schematic model of abiotic (black arrows) and biotic (white arrows) processes inducing corrosion of steel on the mooring chain under deep-sea conditions. In the upper panel, the abiotic production of H_2_ might provide a chemical cue for the initial biofilm colonization

Additionally, thiosulfate S_2_O^2−^_3_ or elemental sulfur S^0^ can be disproportionated into sulfide or sulfur and sulfate by *Desulfocapsa* and certain *Desufovibrio* species (Fig. 6):

3 $${\mathrm{S}}_2{{\mathrm{O}}^{2-}}_3+{\mathrm{H}}_2\mathrm{O}\to {{\mathrm{S}\mathrm{O}}^{2-}}_4\kern0.5em +{\mathrm{H}\mathrm{S}}^{-}+{\mathrm{H}}^{+}$$

4 $${4\mathrm{S}}^0+{4\mathrm{H}}_2\mathrm{O}\to {{\mathrm{SO}}^{2-}}_4+{3\mathrm{HS}}^{-}+{5\mathrm{H}}^{+}$$

The zero-valent sulfur (S^0^) is used by *Sulfurovum* or *Sulfurimonas*, which may also use H_2_ or by disproportionation:

5 $${\mathrm{S}}^0+{\mathrm{H}}_2\to {\mathrm{H}\mathrm{S}}^{-}+{\mathrm{H}}^{+}$$

Moreover, the flux of CO_2_ to the extracellular space (Fig. 5) is likely to further contribute to the formation of FeCO_3_ deposits along with the formation of FeS, which was detected by XRD (Fig. 6) [68].

6 $${4\mathrm{Fe}}^0+{{\mathrm{SO}}_4}^{2-}+{{3\mathrm{HCO}}_3}^{-}+{\mathrm{H}}_2\mathrm{O}\to \mathrm{FeS}+{3\mathrm{FeCO}}_3+{5\mathrm{HO}}^{-}$$

Sulfur cycling microorganisms may gain energy by accelerating the reaction between metallic iron and elemental sulfur, which would take place more slowly in the absence of these microorganisms. FeS, S^0^, and FeCO_3_ were significant components of the mooring chain surface deposit. The conductive properties of these deposits would allow the transfer of electrons, further increasing the corrosion rate [68]. Additionally, genomic evidence showed that there was the potential for the novel thiosulfate oxidation pathway recently described in the deep sea [69] (Supplementary Table S4). Flux-based analysis of MAGs highlighted the secretion of H^+^ and CO_2_ in the mooring chain microbial community, supporting reactions 3‑6. Thus, based on these reactions and the organisms present, we suggest that the unexpected extremely high rate of corrosion was the combined result of a unique, deep-sea-adapted microbial community that drives corrosion in a manner that is distinct from the corrosion cases reported in shallower seas, highlighting the need for more detailed understanding of MIC in these extreme environments.

## Conclusions

The corrosion of mooring chain steel was studied in deep-sea conditions after a period of 10 years. The results obtained from this field study elucidate the corrosion mechanisms under high hydrostatic pressure on microbially induced corrosion in seawater environments.

The results obtained in this research enable the following conclusions to be drawn:

- High corrosion rates and the type of corrosion (deep pitting), together with biological findings, suggest that the corrosion of mooring chain links has been driven to a large extent by biotic processes.
- The microbial community formed on the surface of the chain links was unique and distinct from that on the surrounding sediment, showing that specific microbial taxa were enriched on the steel mooring chain surface.
- The results indicate that deep-sea sulfur cycling microorganisms may gain energy by accelerating the reaction between metallic iron and elemental sulfur, which would take place more slowly in their absence thus causing the high corrosion rate detected. Such an energy-generating strategy might be particularly important in low-energy environments such as the deep sea.

## Supplementary Information

**Additional file 1.**

## Data Availability

The raw sequencing data and the MAGs from this study have been deposited in the National Center for Biotechnology Information under BioProject number PRJNA693108. The SEM images and other experimental data have been deposited in the data repository of Nanyang Technological University (DR-NTU) under the doi 10.21979/N9/TETZEM.

## References

[1] Rajala P, Bomberg M, Huttunen-Saarivirta E, Priha O, Tausa M, Carpén L (2016). Influence of chlorination and choice of materials on fouling in cooling water system under brackish seawater conditions. Materials (Basel).

[2] Rajala P, Sohlberg E, Priha O, Tsitko I, Väisänen H, Tausa M (2016). Biofouling on coated carbon steel in cooling water cycles using brackish seawater. J Mar Sci Eng.

[3] Huttunen-Saarivirta E, Rajala P, Marja-Aho M, Maukonen J, Sohlberg E, Carpén L (2018). Ennoblement, corrosion, and biofouling in brackish seawater: comparison between six stainless steel grades. Bioelectrochemistry.

[4] Yang Y, Zhang T, Shao Y, Meng G, Wang F (2010). Effect of hydrostatic pressure on the corrosion behaviour of Ni-Cr-Mo-V high strength steel. Corros Sci.

[5] Li QS, Luo SZ, Xing XT, Yuan J, Liu X, Wang JH (2019). Effect of deep sea pressures on the corrosion behavior of X65 steel in the artificial seawater. Acta Metall Sin (English Lett).

[6] Wang Z, Cong Y, Zhang T (2014). Effect of hydrostatic pressure on the pitting corrosion behavior of 316L stainless steel. Int J Electrochem Sci.

[7] Sun F, Ren S, Li Z, Liu Z, Li X, Du C (2017). Comparative study on the stress corrosion cracking of X70 pipeline steel in simulated shallow and deep sea environments. Mater Sci Eng A.

[8] Yang Z, Kan B, Li J, Su Y, Qiao L, Volinsky AA. A statistical study on the effect of hydrostatic pressure on metastable pitting corrosion of X70 pipeline steel. Materials (Basel). 2017;10. 10.3390/ma10111307 PMC5706254 29135912

[9] Machuca LL, Bailey SI, Gubner R, Watkin ELJ, Ginige MP, Kaksonen AH. Crevice corrosion of duplex stainless steels in the presence of natural marine biofilms. 2012.

[10] Wu T, Yan M, Zeng D, Xu J, Sun C, Yu C (2015). Stress corrosion cracking of X80 steel in the presence of sulfate-reducing bacteria. J Mater Sci Technol.

[11] Rajala P, Huttunen-Saarivirta E, Bomberg M, Carpén L (2019). Corrosion and biofouling tendency of carbon steel in anoxic groundwater containing sulphate reducing bacteria and methanogenic archaea. Corros Sci.

[12] Fang J, Zhang L, Bazylinski DA (2010). Deep-sea piezosphere and piezophiles: geomicrobiology and biogeochemistry. Trends Microbiol.

[13] Lauro FM, Bartlett DH (2008). Prokaryotic lifestyles in deep sea habitats. Extremophiles.

[14] Rajala P, Bomberg M, Vepsäläinen M, Carpén L (2017). Microbial fouling and corrosion of carbon steel in deep anoxic alkaline groundwater. Biofouling.

[15] Meyer G, Schneider-Merck T, Böhme S, Sand W (2002). A simple method for investigations on the chemotaxis of A. ferrooxidans and D. vulgaris. Acta Biotechnol.

[16] Duan J, Wu S, Zhang X, Huang G, Du M, Hou B (2008). Corrosion of carbon steel influenced by anaerobic biofilm in natural seawater. Electrochim Acta.

[17] Kuang F, Wang J, Yan L, Zhang D (2007). Effects of sulfate-reducing bacteria on the corrosion behavior of carbon steel. Electrochim Acta.

[18] Castaneda H, Benetton XD (2008). SRB-biofilm influence in active corrosion sites formed at the steel-electrolyte interface when exposed to artificial seawater conditions. Corros Sci.

[19] Beech IB, Campbell SA (2008). Accelerated low water corrosion of carbon steel in the presence of a biofilm harbouring sulphate-reducing and sulphur-oxidising bacteria recovered from a marine sediment. Electrochim Acta.

[20] Stipaničev M, Turcu F, Esnault L, Schweitzer EW, Kilian R, Basseguy R (2013). Corrosion behavior of carbon steel in presence of sulfate-reducing bacteria in seawater environment. Electrochim Acta.

[21] Javaherdashti R, Singh Raman RK, Panter C, Pereloma EV (2006). Microbiologically assisted stress corrosion cracking of carbon steel in mixed and pure cultures of sulfate reducing bacteria. Int Biodeterior Biodegrad.

[22] Stipaničev M, Rosas O, Basseguy R, Turcu F (2014). Electrochemical and fractographic analysis of microbiologically assisted stress corrosion cracking of carbon steel. Corros Sci.

[23] ASTM. G 1-90 standard practice for preparing, cleaning, and evaluating corrosion test coupons. 2017. ASTM Standard.

[24] Hartman AL, Lough DM, Barupal DK, Fiehn O, Fishbein T, Zasloff M (2009). Human gut microbiome adopts an alternative state following small bowel transplantation. Proc Natl Acad Sci U S A.

[25] Bano N, Ruffin S, Ransom B, Hollibaugh JT (2004). Phylogenetic composition of arctic ocean archaeal assemblages and comparison with Antarctic assemblages. Appl Environ Microbiol.

[26] Barns SM, Fundyga RE, Jeffries MW, Pace NR (1994). Remarkable archaeal diversity detected in a Yellowstone National Park hot spring environment. Proc Natl Acad Sci.

[27] Geets J, Borremans B, Diels L, Springael D, Vangronsveld J, van der Lelie D (2006). DsrB gene-based DGGE for community and diversity surveys of sulfate-reducing bacteria. J Microbiol Methods.

[28] Wagner M, Roger AJ, Flax JL, Brusseau GA, Stahl DA (1998). Phylogeny of dissimilatory sulfite reductases supports an early origin of sulfate respiration. J Bacteriol.

[29] Wilkins D, Van Sebille E, Rintoul SR, Lauro FM, Cavicchioli R (2013). Advection shapes Southern Ocean microbial assemblages independent of distance and environment effects. Nat Commun.

[30] Gusareva ES, Acerbi E, Lau KJX, Luhung I, Premkrishnan BNV, Kolundžija S (2019). Microbial communities in the tropical air ecosystem follow a precise diel cycle. Proc Natl Acad Sci.

[31] Bolyen E, Rideout JR, Dillon MR, Bokulich NA, Abnet CC, Al-Ghalith GA (2019). Reproducible, interactive, scalable and extensible microbiome data science using QIIME 2. Nat Biotechnol.

[32] Callahan BJ, McMurdie PJ, Rosen MJ, Han AW, Johnson AJ, Holmes SP (2016). DADA2: High-resolution sample inference from Illumina amplicon data. Nat Methods.

[33] Lozupone C, Knight R (2005). UniFrac: a new phylogenetic method for comparing microbial communities. Appl Env Microbiol.

[34] Eren AM, Esen OC, Quince C, Vineis JH, Morrison HG, Sogin ML, et al. Anvi’o: an advanced analysis and visualization platform for ’omics data. PeerJ. 2015:e1319. 10.7717/peerj.1319 PMC4614810 26500826

[35] Li D, Luo R, Liu CM, Leung CM, Ting HF, Sadakane K (2016). MEGAHIT v1.0: a fast and scalable metagenome assembler driven by advanced methodologies and community practices. Methods.

[36] Langmead B, Salzberg SL (2012). Fast gapped-read alignment with Bowtie 2. Nat Methods.

[37] Li H, Handsaker B, Wysoker A, Fennell T, Ruan J, Homer N (2009). The sequence alignment/map format and SAMtools. Bioinformatics.

[38] Hyatt D, Chen GL, Locascio PF, Land ML, Larimer FW, Hauser LJ (2010). Prodigal: prokaryotic gene recognition and translation initiation site identification. BMC Bioinformatics.

[39] Menzel P, Ng KL, Krogh A (2016). Fast and sensitive taxonomic classification for metagenomics with Kaiju. Nat Commun.

[40] Parks DH, Imelfort M, Skennerton CT, Hugenholtz P, Tyson GW (2015). CheckM: assessing the quality of microbial genomes recovered from isolates, single cells, and metagenomes. Genome Res.

[41] Chaumeil PA, Mussig AJ, Hugenholtz P, Parks DH. GTDB-Tk: a toolkit to classify genomes with the Genome Taxonomy Database. Bioinformatics. 2019. 10.1093/bioinformatics/btz848 PMC7703759 31730192

[42] Paradis E, Claude J, Strimmer K (2004). APE: Analyses of phylogenetics and evolution in R language. Bioinformatics.

[43] Oksanen J, Blanchet FG, Friendly M, Kindt R, Legendre P, McGlinn D (2016). Vegan: community ecology package, R Package.

[44] Camacho C, Coulouris G, Avagyan V, Ma N, Papadopoulos J, Bealer K (2009). BLAST+: architecture and applications. BMC Bioinformatics.

[45] Edgar RC (2004). MUSCLE: a multiple sequence alignment method with reduced time and space complexity. BMC Bioinformatics.

[46] Garber AI, Nealson KH, Okamoto A, McAllister SM, Chan CS, Barco RA (2020). FeGenie: a comprehensive tool for the identification of iron genes and iron gene neighborhoods in genome and metagenome assemblies. Front Microbiol.

[47] Johnson M, Zaretskaya I, Raytselis Y, Merezhuk Y, McGinnis S, Madden TL (2008). NCBI BLAST: a better web interface. Nucleic Acids Res.

[48] Zhu W, Lomsadze A, Borodovsky M (2010). Ab initio gene identification in metagenomic sequences. Nucleic Acids Res.

[49] Seemann T (2014). Prokka: rapid prokaryotic genome annotation. Bioinformatics.

[50] Flynn JM, Hubley R, Goubert C, Rosen J, Clark AG, Feschotte C (2020). RepeatModeler2 for automated genomic discovery of transposable element families. Proc Natl Acad Sci U S A.

[51] Arkin AP, Cottingham RW, Henry CS, Harris NL, Stevens RL, Maslov S (2018). KBase: the United States Department of Energy Systems Biology Knowledgebase. Nat Biotechnol.

[52] Brettin T, Davis JJ, Disz T, Edwards RA, Gerdes S, Olsen GJ (2015). RASTtk: a modular and extensible implementation of the RAST algorithm for building custom annotation pipelines and annotating batches of genomes. Sci Rep.

[53] Henry CS, DeJongh M, Best AA, Frybarger PM, Linsay B, Stevens RL (2010). High-throughput generation, optimization and analysis of genome-scale metabolic models. Nat Biotechnol.

[54] Orth JD, Thiele I, Palsson BO (2010). What is flux balance analysis?. Nat Biotechnol.

[55] Darzi Y, Letunic I, Bork P, Yamada T (2018). iPath3.0: interactive pathways explorer v3. Nucleic Acids Res.

[56] Reinhart FM. Corrosion of metals and alloys in the deep ocean. 1976.

[57] Clover D, Kinsella B, Pejcic B, De Marco R (2005). The influence of microstructure on the corrosion rate of various carbon steels. J Appl Electrochem.

[58] Phan H, Wade S, Blackall L (2020). Microbial communities of orange tubercles in accelerated. Appl Env Microbiol.

[59] Takeuchi M, Whillock GOH, Whillock GO (2012). Effect of endgrain attack on corrosion of 18Cr-10Ni austenitic stainless steel in simulated dissolver liquor.

[60] Lee JS, Little BJ (2019). A mechanistic approach to understanding microbiologically influenced corrosion by metal-depositing bacteria. Corrosion.

[61] Miller JD, Warren BJ, Chabot LG, Jenkins JF. Microbiologically influenced corrosion of Gulf of Mexico mooring chain at 6,000 feet depths. Proc. 31st Int. Conf. Ocean. Offshore, Arct. Eng. 2012. p OMAE 2012-84067.

[62] Little BJ, Lee JS, Gerke TL. An introduction to rusticles, accumulated iron oxides/hydroxides, on shipwrecks. Corros 2016 . 2016. NACE International, Vancouver, British Columbia, Canada 6.

[63] Waldemar Finster K, Urup Kjeldsen K, Kube M, Reinhardt R, Mussmann M, Amann R (2013). Complete genome sequence of Desulfocapsa sulfexigens, a marine deltaproteobacterium specialized in disproportionating inorganic sulfur compounds. Stand Genomic Sci.

[64] Finster K, Liesack W, Thamdrup B (1998). Elemental sulfur and thiosulfate disproportionation by Desulfocapsa sulfoexigens sp. nov., a new anaerobic bacterium isolated from marine surface sediment. Appl Environ Microbiol.

[65] Finster K (2008). Microbiological disproportionation of inorganic sulfur compounds. J Sulfur Chem.

[66] Campanaro S, Treu L, Valle G (2008). Protein evolution in deep sea bacteria: an analysis of amino acids substitution rates. BMC Evol Biol.

[67] Mori JF, Scott JJ, Hager KW, Moyer CL, Küsel K, Emerson D (2017). Physiological and ecological implications of an iron- or hydrogen-oxidizing member of the Zetaproteobacteria, Ghiorsea bivora, gen. nov., sp. Nov. ISME J.

[68] Enning D, Venzlaff H, Garrelfs J, Dinh HT, Meyer V, Mayrhofer K (2012). Marine sulfate-reducing bacteria cause serious corrosion of iron under electroconductive biogenic mineral crust. Environ Microbiol.

[69] Zhang J, Liu R, Xi S, Cai R, Zhang X, Sun C (2020). A novel bacterial thiosulfate oxidation pathway provides a new clue about the formation of zero-valent sulfur in deep sea. ISME J.

