# Supplementary material for: Sulfate-dependant microbially induced corrosion of mild steel in the deep sea: a 10-year microbiome study
Source: Microbiome. 2022 Jan 13;10:4. doi: 10.1186/s40168-021-01196-6 (PMC8756651; doi:10.1186/s40168-021-01196-6)

SUPPLEMENTARY MATERIAL

Supplementary Figures S1, S2, S3

Supplementary Tables S1, S2, S3, S4

Supplementary Dataset S1

Figure S1. Phylogenetic trees of the MAGs recovered in this study and closely related taxa. Trees are from the GTDB-Tk results.


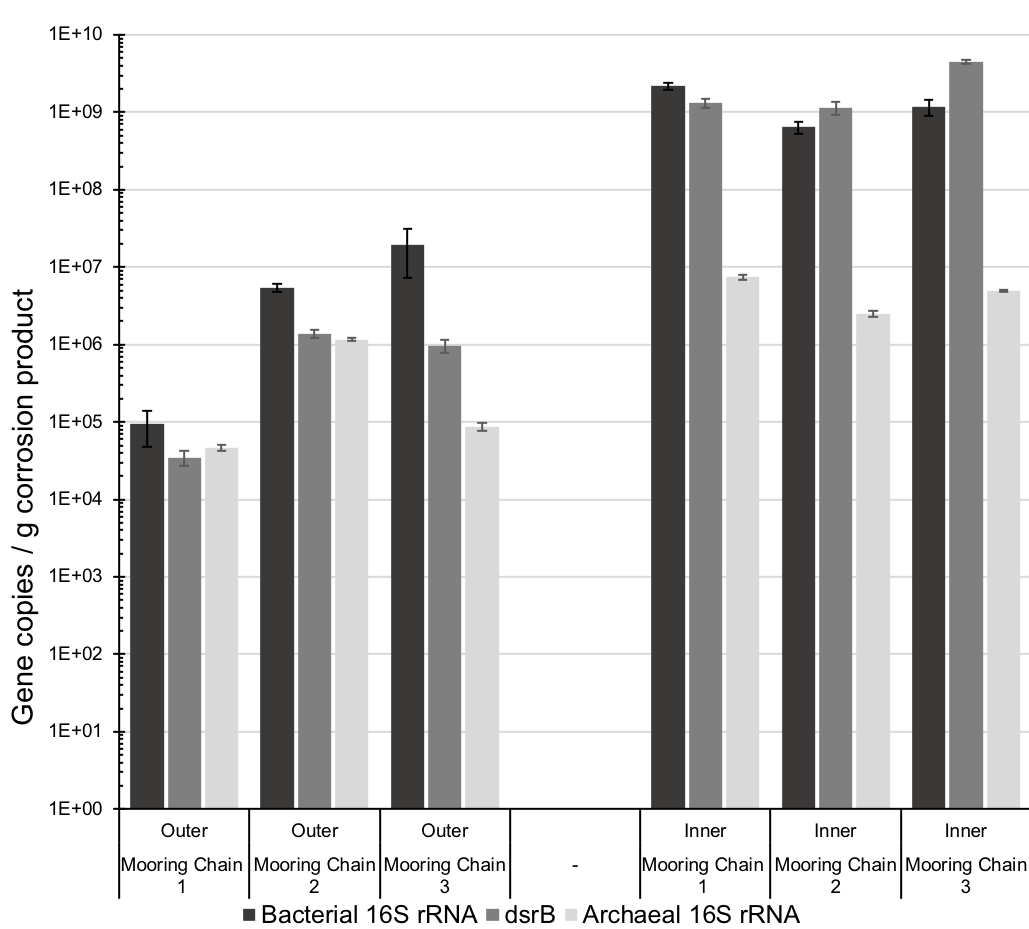


Figure S2. Quantitative PCR analysis of bacterial and archaeal 16S rRNA gene copies and *dsrB* gene copies per gram of corrosion product. Error bars represent the standard deviation of triplicate reactions.

Figure S3 A) Frequency of the length of intergenic regions (IR) from the deep-sea isolate *Desulfocapsa sp.* MC-01 and the closest shallow water relative *Desulfocapsa sulfexigens* DSM 10523. B) The frequency histogram is based on the length of IR from isolate MC-01 and isolate DSM 10523. The lengths of IRs follows a power law distribution. The mean IR length for DSM 10523 was 1447.6bp. The mean IR length for MC-01 was 1015.6bp. MC-01 has a higher rate of shorter IR (1-500bp, 501-1000bp, 1001-1500bp) than DSM_10523. DSM_10523 has a higher rate of longer IR (3001-3500bp, 3501-4000bp, 4001-4500bp, 4501-5000) than DSM_10523.

Table S1. Summary of flux balance analyses using KBase.

Table S2. Comparison of transposable elements (TE) between the deep-sea isolates and the closest shallow water isolates. Orange represents deep-sea isolates. Blue represents shallow water isolates. TEs were identified using RepeatModeler. The number of TEs for each MAG were normalised to the sequence length and compared to the normalised numbers from the closest complete genomes from shallow water. The TEs classified into families which are found in all the taxa under consideration were defined as conserved TEs.

Table S3. Recovery of genes involved in iron metabolism. Green represents the genes recovered in at least one MAG. Red represents the genes that were not present in any of the MAGs. The search was performed using FeGenie

Table S4. Recovery of genes involved in thiosulfate metabolism. Green indicates the presence of that particular gene in a MAG while red represents the absence.

Dataset S1. EDS spectra and elemental composition (weight % and atom %) at selected areas of the corrosion products, highlighted in the SEM image.


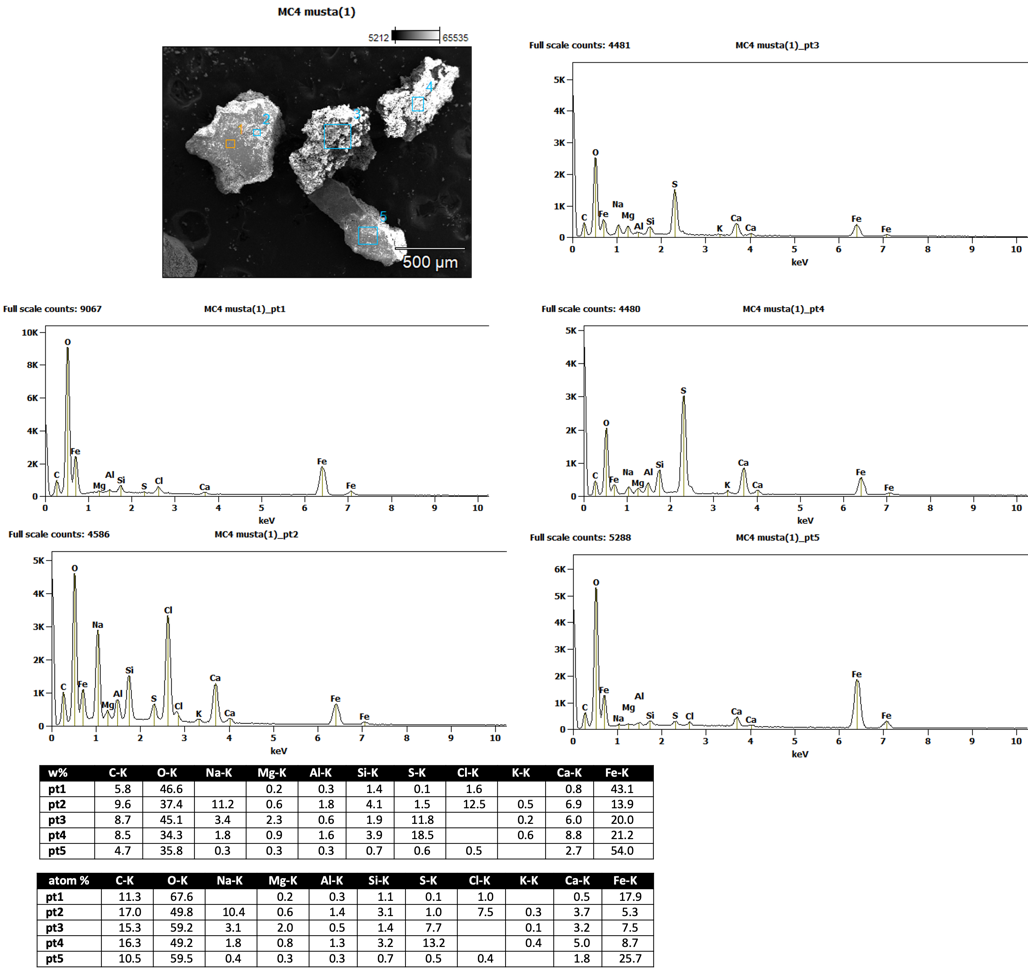


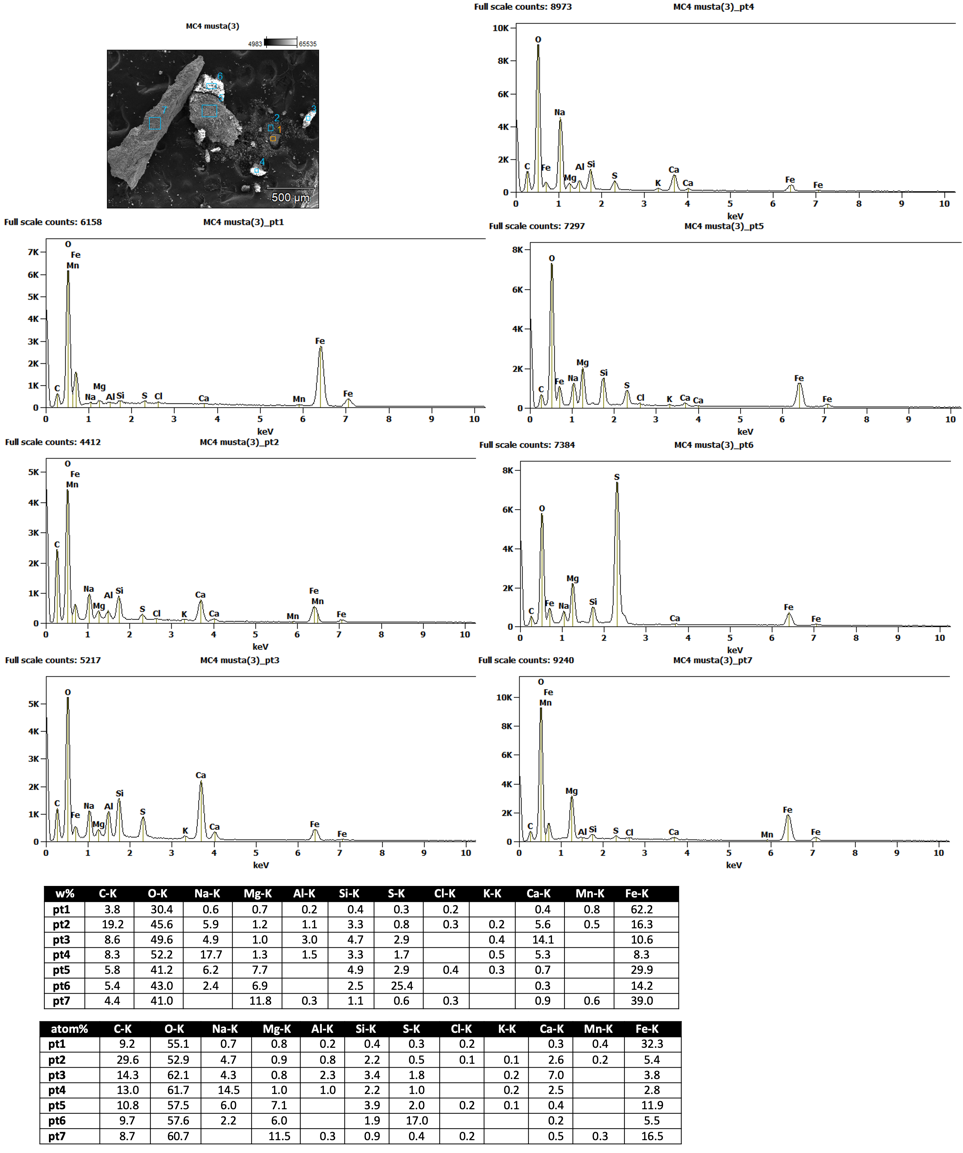

Supplement: Supplementary file 1 — Additional file 1. [file 40168_2021_1196_MOESM1_ESM.docx]
